# Overcoming inter-subspecific hybrid sterility in rice by developing *indica*-compatible *japonica* lines

**DOI:** 10.1038/srep26878

**Published:** 2016-06-01

**Authors:** Jie Guo, Xiaomei Xu, Wentao Li, Wenyin Zhu, Haitao Zhu, Ziqiang Liu, Xin Luan, Ziju Dai, Guifu Liu, Zemin Zhang, Ruizhen Zeng, Guang Tang, Xuelin Fu, Shaokui Wang, Guiquan Zhang

**Affiliations:** 1 State Key Laboratory for Conservation and Utilization of Subtropical Agro-Bioresources, South China Agricultural University, Guangzhou 510642, China

## Abstract

Rice (*Oryza sativa* L.) is an important staple crop. The exploitation of the great heterosis that exists in the inter-subspecific crosses between the *indica* and *japonica* rice has long been considered as a promising way to increase the yield potential. However, the male and female sterility frequently occurred in the inter-subspecific hybrids hampered the utilization of the heterosis. Here we report that the inter-subspecific hybrid sterility in rice is mainly affected by the genes at *Sb*, *Sc*, *Sd* and *Se* loci for F_1_ male sterility and the gene at *S5* locus for F_1_ female sterility. The *indica*-compatible *japonica* lines (ICJLs) developed by pyramiding the *indica* allele (*S*-i) at *Sb*, *Sc*, *Sd* and *Se* loci and the neutral allele (*S*-n) at *S5* locus in *japonica* genetic background through marker-assisted selection are compatible with *indica* rice in pollen fertility and in spikelet fertility. These results showed a great promise of overcoming the inter-subspecific hybrid sterility and exploiting the heterosis by developing ICJLs.

Asian cultivated rice (*Oryza sativa* L.) is the staple food for more than half of the world’s population. Continuing production of high-yielding rice is essential for maintaining global food security^1^,^2^. Asian cultivated rice is mainly distributed in Asia as well as some other areas in the world. During the course of evolution, the cultivated rice was differentiated into two distinct eco-geographic subspecies, *indica* and *japonica*^3^. The *indica* subspecies is generally adapted to the humid regions of tropical and subtropical Asia whereas *japonica* rice is mainly distributed in the temperate regions. Since China pioneered *indica* hybrid rice production in the 1970’s, a great success has been achieved in the hybrid rice development in China and around the world^4^,^5^. The *indica* hybrid rice usually has more about 20% yield increase compared with conventional varieties, and now accounts for more than half of the annual rice planting area in China^5^,^6^. Currently used rice varieties with a close genetic relationship have limited heterosis, leading to a yield plateau for *indica* hybrid rice production^7^. Strong heterosis exists in the inter-subspecific hybrids and the exploitation of this heterosis has long been considered as a promising way to further increase rice yield potential^4^,^5^,^8^. However, the major obstacle of utilizing the heterosis between the subspecies is the strong hybrid sterility^3^,^9^,^10^,^11^,^12^.

The hybrid sterility occurs frequently in many remote crosses in rice^9^. The spikelet fertility of the hybrids varied widely among the crosses from almost completely sterile to fully fertile in a diallel set of 210 crosses involving 21 parents^10^. Spikelet fertility of F_1_ hybrids was found to be negatively related with the genetic divergence index of parental varieties^13^. The genetic basis of the inter-subspecific hybrid sterility has been extensively investigated in the last several decades. Approximately 50 loci for hybrid fertility have been identified in rice, including loci causing female gamete abortion and those inducing pollen sterility. Among the loci for hybrid fertility, some were identified in the inter-subspecific crosses of *O. sativa* while others were found in the crosses between *O. sativa* and other species of *Oryza* genus^11^. Among the loci causing female sterility in the inter-subspecific hybrids, *S5* is a major locus^14^,^15^. The *S5* locus was mapped onto chromosome 6 and three alleles, namely *indica* allele (*S5*-i), *japonica* allele (*S5*-j) and neutral allele (*S5*-n), were identified at the locus^14^,^16^. The *S5* gene was subsequently isolated through map-based cloning approach, which encodes an aspartic protease to manipulate the female fertility of the hybrids^17^. The *S5*-i and *S5*-j alleles differed only at two nucleotides for the female sterility, while the *S5*-n allele has a loss-function mutation with 136 bp DNA-sequence deletion for the compatibility^17^,^18^,^19^. For F_1_ pollen sterility, five loci, i.e. *Sa* (*S-E3*), *Sb* (*S-E2*), *Sc* (*S-E5*), *Sd* and *Se*, were identified through a series of allelic test-crosses^20^,^21^,^22^,^23^,^24^. Among five identified F_1_ pollen sterility loci, the *Sa* locus was mapped to a region of 30 kb on chromosome 1^25^,^26^, and was subsequently cloned^27^; the *Sb* locus was delimited to a region of 27 kb on chromosome 5^28^,^29^; the *Sc* locus was narrowed down to a region of 46 kb on chromosome 3^30^,^31^; the *Sd* locus was delimited to a region of 67 kb on chromosome 1^32^; and the *Se* locus was mapped to an interval of 5 cM on chromosome 12^33^. Interestingly, some loci for hybrid sterility, such as *S24* and *S31*^34^,^35^, *S35*^36^, *S25* and *S36*^37^,^38^ were also identified in the same regions as the *Sb*, *Sd* and *Se* loci, respectively, by different research groups.

To overcome the inter-specific hybrid sterility, a strategy of developing *indica*-compatible *japonica* lines (ICJLs) which carry *S*-i alleles at the loci for hybrid sterility in *japonica* genetic background, was proposed^23^,^39^. The pyramiding effect of the genes for hybrid sterility was evaluated by developing pyramiding lines with *S*-i alleles at multiple loci of *Sa*, *Sb* and *Sc*^24^. Here, we report that the inter-subspecific hybrid sterility can be overcome by using ICJLs. The ICJLs carrying the *S*-i alleles at *Sb*, *Sc*, *Sd* and *Se* loci for F_1_ pollen fertility and the *S*-n allele at *S5* locus for F_1_ female fertility in *japonica* genetic background are compatible with *indica* while incompatible with *japonica*, reversing the compatibility of general *japonica* rice. Our results showed that the ICJLs would be an important genetic stock for overcoming the inter-specific hybrid sterility in rice.

## Results

### F_1_ pollen sterility caused by allelic interaction at the *Sb*, *Sc*, *Sd* and *Se* loci

**Figure 1:**
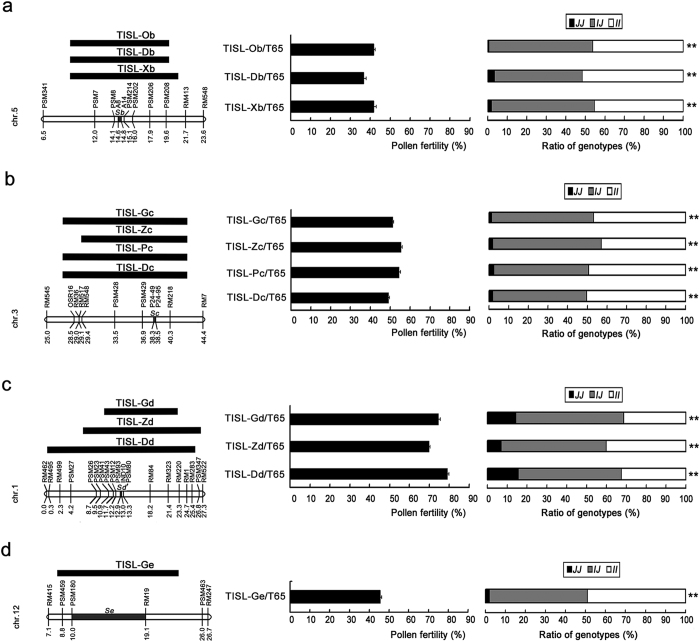
Location and genetic effect of the genes at the *Sb*, *Sc*, *Sd* and *Se* loci on the chromosomal substituted segments in the TISLs. (**a**) The *Sb* locus in TISL-Ob, TISL-Db and TISL-Xb. (**b**) The *Sc* locus in TISL-Gc, TISL-Zc, TISL-Pc and TISL-Dc. (**c**) The *Sd* locus in TISL-Gd, TISL-Zd and TISL-Dd. (**d**) The *Se* locus in TISL-Ge. *Left*: Chromosome locations of the *Sb*, *Sc*, *Sd* and *Se* loci on the substituted segments in the TISLs. The black horizontal bars represent substituted segments in the TISLs. The positions of the *Sb*, *Sc*, *Sd* and *Se* loci are indicated in the chromosome maps. *Middle*: Pollen fertility of F_1_ hybrids from the crosses of TISLs with T65. Error bars represent the SD. *Right*: Ratios of genotypes at the *Sb*, *Sc*, *Sd* and *Se* loci in the F_2_ populations from the crosses of TISLs with T65. *JJ*, Genotype of T65 (*S*-j/*S*-j). *IJ*, Heterozygous genotypes (*S*-i/*S*-j). *II*, Genotypes of TISLs (*S*-i/*S*-i). **Significantly different at the 0.01 probability level.

To assess F_1_ pollen sterility caused by the loci for F_1_ pollen sterility, a set of Taichung65 (T65) isogenic F_1_-sterile lines (TISLs) with the *indica* allele at the locus for F_1_ pollen sterility in the genetic background of T65 (a *japonica* variety) were developed by marker-facilitated backcrossing (Supplementary information). Each TISL carried only one chromosomal substituted segment from a donor *indica* cultivar detected by a survey of the genome with molecular markers (Fig. 1, Supplementary Fig. 1). Of the 11 TISLs, 3, 4, 3 and 1 TISLs carried *S*-i at the loci *Sb*, *Sc*, *Sd* and *Se*, respectively (Fig. 1, Supplementary Table 1). All the TISLs were test-crossed with T65 which has *S*-j allele at the all loci to generate heterozygote *S*-i/*S*-j at the corresponding locus. The F_1_ hybrids in the crosses showed partial pollen fertility, ranging from 36.59% to 79.00%. In all the crosses, distorted segregation from the Mendelian segregation ratio was observed in the F_2_ populations, in which the ratio of genotype JJ (*S*-j/*S*-j) was significantly lower than that of genotype II (*S*-i/*S*-i) (Fig. 1, Supplementary Table 2). The results indicated that the interaction between the *S*-i allele from the TISLs and the *S*-j allele from T65 at the loci caused pollen with the *S*-j allele aborted in varying degrees in the crosses. Similar results were obtained from the test-crosses of TISLs with other *japonica* testers (Supplementary Table 3).

### Pyramiding effect of the genes for F_1_ pollen sterility at the *Sb*, *Sc*, *Sd* and *Se* loci

**Figure 2:**
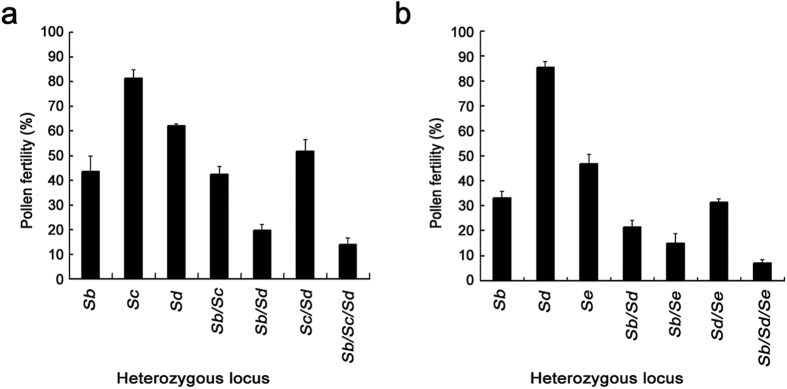
F_1_ pollen sterility caused by allelic interactions at different combinations of the *Sb*, *Sc*, *Sd* and *Se* loci. (**a**) F_1_ pollen fertility in the crosses of TISLs developed from the Dijiaowujian donor with T65 in the first cropping season in 2002. (**b**) F_1_ pollen fertility in the crosses of TISLs developed from the Guangluai4 donor with T65 in the second cropping season in 2005. Error bars represent the SD.

To analyse the pyramiding effect of the genes for F_1_ pollen sterility at the *Sb*, *Sc*, *Sd* and *Se* loci, a set of 7 TISLs with different numbers of *S*-i allele at the *Sb*, *Sc* and *Sd* loci were developed using the *indica* variety Dijiaowujian as the *S*-i allele donor. In the test-crosses of the TISLs with T65, the F_1_ pollen fertility was 43.5%, 81.3% and 62.0% in the genotypes of *S*-i/*S*-j at the single locus of *Sb*, *Sc* and *Sd*, respectively. The pollen fertility was 42.4%, 19.7% and 51.7% in the double heterozygotes at *Sb* and *Sc*, *Sb* and *Sd*, and *Sc* and *Sd*, respectively, and the fertility was only 14.0% in the trihybrid at the *Sb*, *Sc* and *Sd* loci (Fig. 2a). These results indicated that the pollen fertility of heterozygotes decreased with the increase of the number of heterozygous loci for the F_1_ pollen sterility, and the pollen fertility of heterozygotes at multiple loci was approximate to the product of pollen fertility in heterozygotes at each of the loci. Similar results were observed in a BC_4_F_2_ population derived from the backcross between Guangluai4 (a donor of the *S*-i allele at the *Sb*, *Sd* and *Se* loci) and T65. The F_1_ pollen fertility was 33.1%, 85.4% and 46.8% for the single heterozygous locus, respectively, whereas it was 21.3%, 15.0% and 31.3% for the two heterozygous loci of *Sb* and *Sd*, *Sb* and *Se*, and *Sd* and *Se*, respectively, and the fertility was only 7.0% for the line with three heterozygous alleles at *Sb*, *Sd* and *Se* loci (Fig. 2b).

### Overcoming the pollen sterility of the inter-subspecific hybrids by developing of the TISLs with *S*-i alleles at the *Sb*, *Sc*, *Sd* and *Se* loci

**Figure 3:**
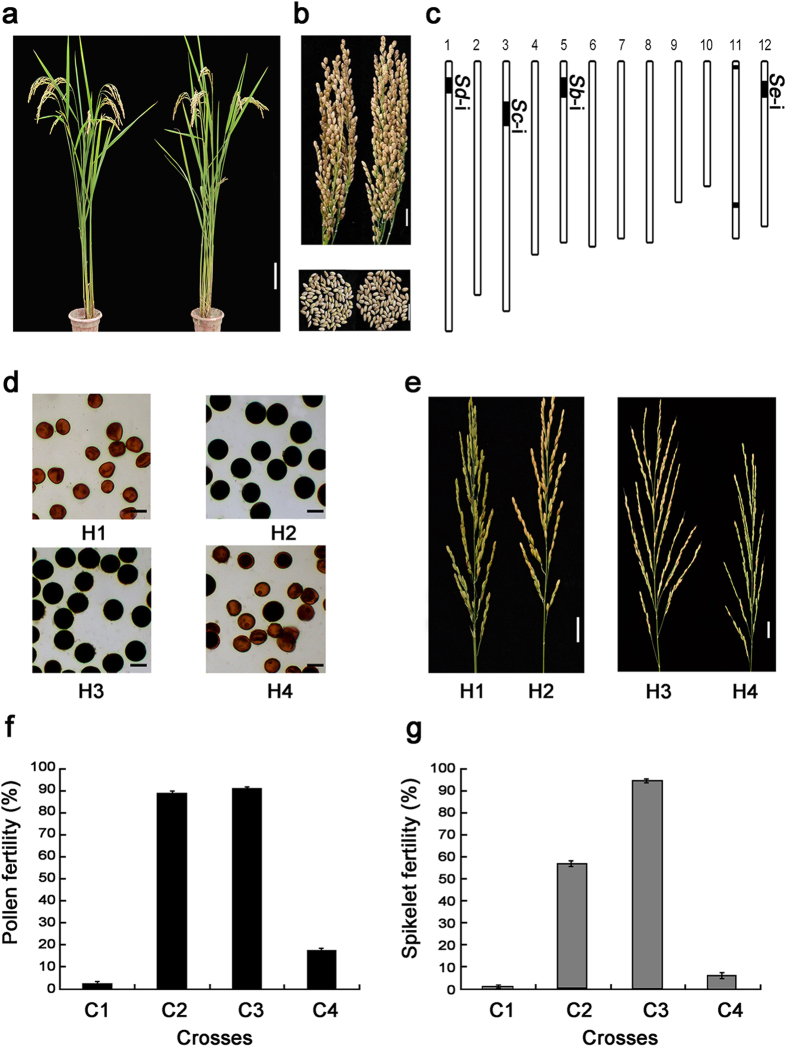
Phenotypes and genotypes of TISL-Dbc-Gde with *S*-i at the *Sb*, *Sc*, *Sd* and *Se* loci. (**a**) Plant morphology of TISL-Dbc-Gde (left) and T65 (right). Scale bar, 10 cm. (**b**) Panicle-type and grain-type of TISL-Dbc-Gde (left) and T65 (right). Scale bar, 2 cm. (**c**) Positions of the substituted segments in the genome of TISL-Dbc-Gde. *Vertical* bars are a graphical representation of chromosomes. *Deep* parts are substitution segments from donors, and *Light* parts are the genetic background from T65. (**d**) Pollen grains stained by I_2_-KI solution in the F_1_ hybrids of four crosses. Scale bar, 30 μm. (**e**) Spikelet fertility in the panicles of the F_1_ hybrids of four crosses. Scale bar, 2 cm. (**f**) Pollen fertility of the F_1_ hybrids from the crosses between TISL-Dbc-Gde and the testers in the second cropping season of 2012 (n = 15). (**g**) Spikelet fertility of the F_1_ hybrids from the crosses between TISL-Dbc-Gde and the testers in the second cropping season of 2012 (n = 15). H1, TISL-Dbc-Gde/Ballila; H2, T65/Ballila; H3, TISL-Dbc-Gde/Aijiaonante; H4, T65/Aijiaonante; C1, TISL-Dbc-Gde/*japonica* testers; C2, TISL-Dbc-Gde/*indica* testers; C3, T65/*japonica* testers; C4, T65/*indica* testers. Error bars represent the SD.

TISL-Dbcd with *S*-i at the *Sb*, *Sc* and *Sd* loci from Dijiaowujian and TISL-Gde with *S*-i at the *Sd* and *Se* loci from Guangluai4 were selected to develop pyramiding line with *S*-i at the *Sb*, *Sc*, *Sd* and *Se* loci. The pyramiding line TISL-Dbc-Gde carried the *S*-i allele at the *Sb* and *Sc* loci from Dijiaowujian and the *S*-i allele at the *Sd* and *Se* loci from Guangluai4 (Fig. 3a–c, Supplementary Table 4). TISL-Dbc-Gde carried 62.1 cM of substituted segments with the *S*-i allele from the *indica* donors that accounted for only 4.06% of the rice genome (Fig. 3c, Supplementary Table 4). TISL-Dbc-Gde was similar to T65 in terms of plant type, panicle type and grain type (Fig. 3a,b). No significant difference between TISL-Dbc-Gde and T65 was detected on the traits of days from sowing to heading, plant height, thousand grain weight, grain length, grain width, and panicle number per plant (Supplementary Table 5). These results indicated that TISL-Dbc-Gde was a *japonica* line with a genotype and phenotype similar to T65. To evaluate the compatibility, TISL-Dbc-Gde was test-crossed with a set of testers consisting of typical *indica* and *japonica* varieties for two years. In the F_1_ hybrids from the crosses of TISL-Dbc-Gde with the *japonica* testers, the pollen fertility was 0.97% and 2.40% and the spikelet fertility was close to complete sterility in the two experiments. In the F_1_ hybrids from the crosses of TISL-Dbc-Gde with the *indica* testers, the pollen fertility was 88.22% and 89.17% and the spikelet fertility was 61.25% and 57.15%. As a control, the pollen fertility was 91.40% and 17.83% and the spikelet fertility was 94.60% and 6.96% in the F_1_ hybrids from the crosses of T65 with the *japonica* and *indica* testers, respectively (Fig. 3d–g, Supplementary Tables 6 and 7). These results indicated that TISL-Dbc-Gde showed reverse compatibility with T65. In other words, TISL-Dbc-Gde was compatible with the *indica* testers but incompatible with the *japonica* testers in terms of pollen fertility. Thus, the pollen sterility of the inter-subspecific hybrids was effectively overcome in the crosses of TISL-Dbc-Gde with the *indica* testers.

### Overcoming the spikelet sterility of the inter-subspecific hybrids by developing ICJLs

**Figure 4:**
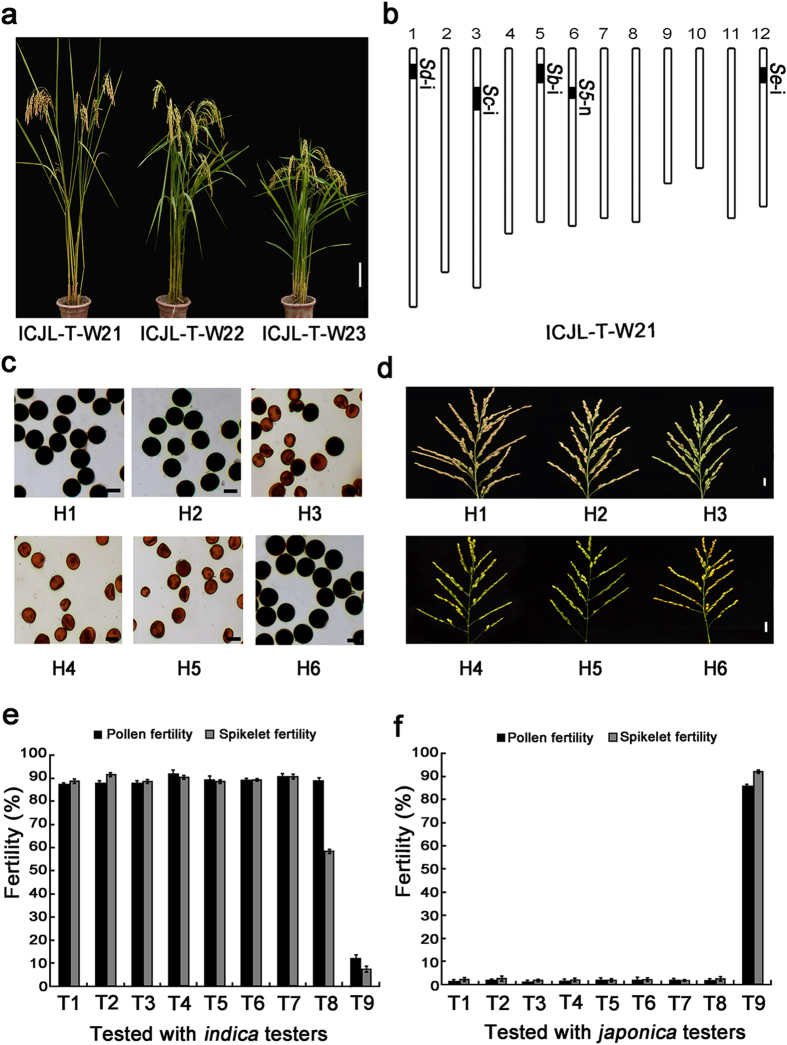
Phenotypes and genotypes of ICJLs with *S*-i at the *Sb*, *Sc*, *Sd* and *Se* loci and *S*-n at the *S5* locus. (**a**) Plant morphologies of ICJL-T-W21, ICJL-T-W22 and ICJL-T-W23. Scale bar, 10 cm. (**b**) Positions of the substituted segments in the ICJL-T-W21 genome. *Vertical* bars are a graphical representation of the chromosomes. *Deep* parts are substitute segments from donors, and *light* parts are the genetic background from *japonica* donors. (**c**) Pollen grains of the F_1_ hybrids from six crosses. Pollen grains were stained with the I_2_-KI solution. Scale bar, 30 μm. (**d**) Spikelet fertility in the panicles of the F_1_ hybrids from six crosses. Scale bar, 2 cm. (**e**) Pollen fertility and spikelet fertility of the F_1_ hybrids from the crosses of ICJLs (TISL-Dbc-Gde and T65 as controls) with the *indica* testers in the second cropping season in 2014 (n = 10). (**f** ) Pollen fertility and spikelet fertility of the F_1_ hybrids from the crosses of ICJLs (TISL-Dbc-Gde and T65 as controls) with the *japonica* testers in the second cropping season in 2014 (n = 10). H1, ICJL-T-W21/9311; H2, TISL-Dbc-Gde/9311; H3, T65/9311; H4, ICJL-T-W21/T65; H5, TISL-Dbc-Gde/T65; H6, T65. T1, ICJL-T-W6; T2, ICJL-T-W19; T3, ICJL-T-W21; T4, ICJL-T-W22; T5, ICJL-T-W23; T6, ICJL-T-W24; T7, ICJL-T-W27; T8, TISL-Dbc-Gde; T9, T65. Error bars represent the SD.

Although the pollen fertility was almost normal, the spikelet fertility was still low in the F_1_ hybrids from the crosses of TISL-Dbc-Gde with the *indica* testers (Supplementary Tables 6 and 7). To survey the problem, the genetic behaviour of six loci for hybrid sterility was investigated in all available crosses of TISL-Dbc-Gde with the *indica* testers. No distorted segregation was found at the *Sa, Sb*, *Sc*, *Sd* and *Se* loci for F_1_ pollen sterility in eighteen F_2_ populations, except for two populations at the *Sa* locus and one population at the *Sd* locus. However, distorted segregation was found in all five F_2_ populations at the *S5* locus for F_1_ embryo sac sterility (Supplementary Table 8). These results implied that the partial spikelet sterility in the crosses of TISL-Dbc-Gde with the *indica* testers was mainly controlled by the allelic interaction of *S*-i/*S*-j at the *S5* locus. To identify the *S5*-n gene, 171 accessions of *O*. *sativa* collected throughout the world were used to identify the allele types at the *S5* locus using functional molecular markers of the *S5* gene. Seventeen of the accessions were identified as carrying the *S5*-n allele (Supplementary Table 9). To develop the ICJLs, seven *japonica* accessions with the *S5*-n allele were selected as donors and crossed with TISL-Dbc-Gde. Totally seven ICJLs from the seven crosses were developed through marker-assisted backcrossing (Supplementary information). The ICJLs carried homozygous *S*-i allele at the *Sb*, *Sc*, *Sd* and *Se* loci from TISL-Dbc-Gde and the *S5*-n allele from the *S5*-n donors (Fig. 4a,b, Supplementary Table 10). To evaluate the compatibility, the ICJLs were test-crossed with a set of testers consisting of typical *indica* and *japonica* varieties. The F_1_ pollen fertility of the ICJLs was similar to that of TISL-Dbc-Gde when test-crossed with the *indica* or *japonica* testers. All of the ICJLs were compatible with the *indica* testers but incompatible with the *japonica* testers in terms of pollen fertility (Fig. 4c,e,f, Supplementary Tables 11 and 12). The F_1_ spikelet fertility of the ICJLs was different from that of TISL-Dbc-Gde when test-crossed with the *indica* testers. All of the ICJLs were compatible with the *indica* testers and had high spikelet fertility in the crosses while they were incompatible with the *japonica* testers and had high spikelet sterility in the crosses (Fig. 4d–f, Supplementary Tables 11 and 13). These results indicated that the ICJLs showed reverse compatibility with T65 and other *japonica* varieties since they were compatible with *indica* while incompatible with *japonica* rice. Thus, the spikelet sterility of the inter-subspecific hybrids was effectively overcome.

## Discussion

Hybrid sterility is the most common form of postzygotic reproductive isolation in plants. The best-known example is perhaps the hybrid sterility between *indica* and *japonica* subspecies of Asian cultivated rice^40^. The hybrid sterility between *indica* and *japonica* subspecies of Asian cultivated rice is a complex trait. It consists of male sterility or pollen sterility and female sterility or embryo-sac sterility, and varies in different crosses and in different environments^15^,^24^,^40^,^41^. In some studies, the hybrid sterility was only detected by spikelet fertility. Thus, it is not known to what extent male and female gamete abortions influence the spikelet fertility^15^. Among approximate 50 loci for the hybrid sterility identified in rice, some loci were detected in inter-subspecific hybrids of *O. sativa* while others were found in inter-specific hybrids between *O. sativa* and other species of *Oryza* genus^11^. Thus, it is not clear how many loci involving in the inter-specific hybrid sterility. In this study, the ICJLs, carrying the *S*-i allele at the *Sb*, *Sc*, *Sd* and *Se* loci and the *S*-n allele at the *S5* locus, were almost completely compatible with *indica* testers and incompatible with *japonica* testers in pollen fertility and in spikelet fertility. These results indicated that the hybrid sterility in inter-subspecific hybrids was mainly controlled by the genes at the *Sb*, *Sc*, *Sd*, *Se* and *S5* loci.

Understanding the genetic basis of hybrid sterility in rice lays the foundation for overcoming the hybrid sterility in inter-subspecific hybrids. At least three strategies have been proposed for overcoming the hybrid sterility in *indica*-*japonica* crosses. First, the neutral alleles, or wide-compatibility genes (WCGs) can be introgressed from the wide-compatibility varieties (WCVs) into the parents whose hybrids exhibit strong yield heterosis. The second strategy is to breed ‘ICJLs’ by introgressing *indica* alleles of several hybrid sterility loci into *japonica* lines by backcrossing. The third is to produce artificial neutral alleles by suppressing expression of the genes causing hybrid sterility with RNAi or microRNA technology, if such gene silencing does not affect the plant growth or development^40^. The *S5*-n gene was identified as a WCG and the varieties carrying *S5*-n, named WCVs, were believed to be compatible with both *indica* and *japonica*^14^. However, it has been frequently found that the *S5*-n gene alone is not sufficient for producing *indica*-*japonica* hybrids with normal fertility. *S5*-n can only overcome the sterility caused by embryo sac abortion^15^. We proposed the strategy of development of ‘ICJLs’ which carry the *S*-i alleles at the loci for hybrid sterility in the *japonica* genetic background by backcrossing and MAS^23^,^39^. Following this strategy, we developed a set of the ICJLs which carry the *S*-i allele at the *Sb*, *Sc*, *Sd* and *Se* loci and the *S*-n allele at the *S5* locus, as an outcome of two decades of continuing pursuit. The ICJLs are compatible with *indica* while incompatible to *japonica* rice. Thus, the hybrid sterility of *indica*-*japonica* rice was effectively overcome in the crosses of the ICJLs with the *indica* rice. It provides the first example of overcoming hybrid sterility in plants.

The breeding of *indica*-*japonica* rice has been practiced since the last decades. On the one hand, the utilization of inter-subspecific crosses has been considered as an efficient approach to develop traditional varieties with high yield potential^1^,^2^. On the other hand, the hybrids between *indica* and *japonica* varieties often show strong heterosis compared with intra-subspecific hybrids. For example, in China’s “super” rice breeding, the two-line or three-line method was used to develop F_1_ hybrid combinations by crossing an intermediate type between *indica* and *japonica* with an *indica* parent in order to use inter-subspecific heterosis^1^,^5^. The ICJLs will be an important genetic stock for breeding of *indica*-*japonica* rice. First, the ICJLs will be used to develop traditional varieties with higher yield potential. In the crosses of ICJLs with *indica* varieties, recombination of the genes from *indica* and *japonica* (ICJLs) will be no longer limited by hybrid sterility. Second, the ICJLs will be used to develop inter-subspecific hybrid rice. In the two-line or three-line rice hybrid system, ICJLs can be used to develop sterile line (A line), or restorer line (R line). Recently, a platform for breeding by design of CMS sterile lines based on an SSSL library in rice was developed^42^. By the combination of the strategy of the ICJLs with the platform for breeding by design, the three-lines of ICJLs for hybrid rice will be developed effectively. It will lead to open a new horizon in utilization of heterosis in the *indica*-*japonica* hybrid rice.

## Methods

### Plant materials and growth conditions

All of the TISLs, ICJLs, *indica* testers, *japonica* testers and other plant materials in this study (Supplementary information) were planted in an experimental station at South China Agricultural University, Guangzhou (23°07′N, 113°15′E). The materials were planted in two cropping seasons each year. The first cropping season was from late February to middle July, and the second cropping season was from late July to middle November. The seeds were sowed on seed beds, and the seedlings were transplanted to the fields. Field management, including irrigation, fertilizer application and pest control, followed essentially normal agricultural practices.

### Marker development and assay

DNA was extracted from fresh young leaves using the CTAB method^43^. The PCR profile used for amplification followed a previously described protocol^44^. The SSR markers used in this study were selected on rice microsatellite maps^45^,^46^. To identify the genotypes at the *S5* locus, we selected five functional markers and designed several markers based on the DNA sequences of *S5* locus^12^,^19^,^47^. To identify the genotypes at the *Sa* locus, we selected marker G02-148^27^. To conduct marker-assisted selection at other loci, SSR markers PSM8, PSM12 and PSM180 and the insertion/deletion (InDel) markers IND19 and ID5 were developed in our lab (Supplementary Table 14).

### Examination of pollen and spikelet fertility

To examine pollen fertility, 6–9 mature flowers were collected from the upper one-third of the panicles of plants during the flowering time and fixed in FAA solution (ethanol, formaldehyde and acetic acid at a ratio of 89:6:5). The pollen was stained with a 1% I_2_-KI solution containing 0.1% (w/v) iodine and 1% (w/v) potassium iodide. More than 300 pollen grains were randomly scanned per plant. The pollen was divided into three types: normal pollen (normal size and fully stained), stained abortive pollen (small size and lightly stained) and empty abortive pollen (small size and empty)^20^. To examine spikelet fertility, three panicles per plant were harvested during the maturation time. Ten to twenty plants were recorded for each variation.

### Statistical analysis

The statistical model *y*_*ij*_ = *μ* + *G*_*i*_ + *ε*_*ij*_ was used to analyse the variance (ANOVA) of all data obtained from the experimental materials in one environment, where *y*_*ij*_ was the *j*th observed value of the target trait for the *i*th genotype and *μ*, *G* and *ε* were the population mean value, genotypic effects and residual error, respectively. For the estimation of genotypic effects on target traits, the significance of the difference between one genotype and the control selected at the *α* probability level was tested by the least significant difference (LSD) method. Data were given as the mean ± standard deviation (SD) and transferred by arcsin^−1^ prior to analysis if they were provided as a percentage. Statistical analysis and graphing were performed using SPSS version 18^48^ and SigmaPlot^49^.

## Additional Information

**How to cite this article**: Guo, J. *et al.* Overcoming inter-subspecific hybrid sterility in rice by developing *indica*-compatible *japonica* lines. *Sci. Rep.* **6**, 26878; doi: 10.1038/srep26878 (2016).

## Supplementary Material

Supplementary Information
