# Supplementary material for: Overcoming inter-subspecific hybrid sterility in rice by developing indica-compatible japonica lines
Source: Sci Rep. 2016 Jun 1;6:26878. doi: 10.1038/srep26878 (PMC4887987; doi:10.1038/srep26878)
Supplement: Supplementary Information [file srep26878-s1.pdf]

Overcoming inter-subspecies hybrid sterility by developing *indica*-compatible *japonica* lines in rice

Jie Guo[1], Xiaomei Xu[1], Wentao Li[1], Wenyin Zhu[1], Haitao Zhu, Ziqiang Liu, Xin Luan, Ziju Dai, Guifu Liu, Zemin Zhang, Ruizhen Zeng, Guang Tang, Xuelin Fu, Shaokui Wang, Guiquan Zhang*

Supplementary information

Development of the TISLs

Two Taichung65 (T65) isogenic sterile lines (TISLs), TISL-Ob and TISL-Pc, were provided by Oka in 1988, who named the two TISLs as E2 and E5 respectively. The TISL-Ob (E2) and TISL-Pc (E5) were developed by the use of T65 as recipient and the *indica* cultivars, Oluenchung and Pehku, as donors respectively, through backcrossing continuously[1]. The TISL-Ob and TISL-Pc were identified to carry *indica* allele (*S*-i) at the loci of *Sb* (*S-E2*) and *Sc* (*S-E5*), respectively[2-3]. The other nine TISLs were developed by the use of T65 as recipient and four *indica* cultivars, Xiobaidao, Dijiaowujian, Zaiyeqing and Guangluai4, as donors by backcrossing and marker-assisted selection. The donors contributing of *S*-i allele at the loci for F_1 pollen sterility were selected based on the results of previous researches[2-3]. The chromosomal substituted segments from the donor in the TISLs were detected by survey of the genome with 277 polymorphic molecular markers distributed on 12 chromosomes of rice (**Supplementary Fig. 1**). The eleven TISLs were used to test the F_1 pollen sterility caused by allelic interaction at the *Sb*, *Sc*, *Sd* and *Se* loci in 2011 (**Supplementary Tables 2-3**).

In the previous study, the *indica* varieties, Dijiaowujian and Guangluai4, were identified to carry *S*-i alleles at the *Sb*, *Sc*, *Sd* and *Se* loci[3]. Thus, Dijiaowujian and Guangluai4 were selected as donors to develop the sets of TISLs by crossing and backcrossing with the T65 recipient through marker-facilitated backcrossing method.

The set of TISLs developed from the Dijiaowujian donor included three TISLs with single locus of *Sb*, *Sc* or *Sd*, three TISLs with two loci of *Sb* and *Sc*, *Sb* and *Sd*, or *Sc* and *Sd*, and one TISL with three loci of *Sb*, *Sc* and *Sd*, on their substituted segments under the T65 genetic background. Another set of TISLs developed from the Guangluai4 donor included three TISLs with single locus of *Sb*, *Sd* or *Se*, three TISLs with two loci of *Sb* and *Sd*, *Sb* and *Se*, or *Sd* and *Se*, and one TISL with three loci of *Sb*, *Sd* and *Se*, on their substituted segments under the T65 genetic background. The two sets of TISLs carrying various number of *S*-i alleles at the loci for F_1 pollen sterility were used to test additive effect of the genes for F_1 pollen sterility at the *Sb*, *Sc*, *Sd* and *Se* loci in 2002 and 2005 (**Fig. 2**).

From the two sets of TISLs developed by the use of Dijiaowujian and Guangluai4 as donors, TISL-Dbcd with *S*-i alleles at the *Sb*, *Sc* and *Sd* loci from Dijiaowujian, and TISL-Gde with *S*-i alleles at the *Sd* and *Se* loci from Guangluai4 were selected to pyramid *S*-i alleles at the *Sb*, *Sc*, *Sd* and *Se* loci. TISL-Dbcd was crossed with TISL-Gde. In segregating populations from the cross of TISL-Dbcd/TISL-Gde, the plants carrying *S*-i alleles at the *Sb*, *Sc*, *Sd* and *Se* loci was selected after surveying the substituted segments with molecular markers. In the pyramiding line, the substituted segments carrying the *Sb* and *Sc* genes were from Dijiaowujian, and the substituted segments carrying the *Sd* and *Se* genes were from Guangluai4. Thus, the pyramiding line was named TISL-Dbc-Gde (**Fig. 3, Supplementary Tables 4-5**). The pyramiding line, TISL-Dbc-Gde, was used to test the effect of overcoming of the pollen sterility in inter-subspecific hybrids in 2011-2012 (**Supplementary Tables 6-7**).

Development of the ICJLs

For developing of the *indica*-compatible *japonica* lines (ICJLs), seven *japonica* accessions, Katy (W6), Kyeema (W19), IR65598-112-2 (W21), Khazar (W22), Lemont (W23), Starbonnet99 (W24), and IAPAR9 (W27), with the *S5*-n gene were selected to use as donors of the *S5*-n gene to overcome the F_1 embryo sac sterility. The seven *japonica* accessions were also used as the donors of chromosomal substituted segments in the library of single segment substitution lines (SSSLs) in the Huajingxian74 genetic background[4-5]. Genome resequencing of twenty-nine parents in the library showed that the seven donors of ICJLs were belong to *japonica* group (data not published). The seven *japonica* accessions were crossed with TISL-Dbc-Gde, respectively. After backcrossing one time by TISL-Dbc-Gde and MAS, seven of ICJLs were developed (**Fig. 4, Supplementary Table 10**). The seven ICJLs were used to test the compatibility with *indica* testers and *japonica* testers in 2014 (**Supplementary Tables 11-13**).

Reference

1. Oka, H.I. Analysis of genes controlling F_1 sterility in rice by the use of isogenic lines. *Genetics* **77,** 521-534 (1974).
2. Zhang, G. & Lu, Y. Genetic studies of the hybrid sterility in cultivated rice (*Oryza sativa*). II. A genic model for F_1 pollen sterility (in Chinese with English abstract). *Acta Genet Sin* **20,** 222-228 (1993).
3. Zhang, G., Lu, Y., Zhang, H., Yang, J. & Liu, G. Genetic studies on the hybrid sterility in cultivated rice (*Oryza sativa*). IV. Genotypes for F_1 pollen sterility (in

Chinese with English abstract). *Acta Genet Sin* **21,** 34-41 (1994).

4. Zhang, G. *et al*. The construction of a library of single segment substitution lines in rice (*Oryza sativa* L.). *Rice Genet Newslett* **21,** 85-87 (2004).
5. Xi, Z. *et al*. Development of a wide population of chromosome single-segment substitution lines in the genetic background of an elite cultivar of rice (*Oryza sativa* L.). *Genome* **49,** 476-484 (2006).

Supplementary tables

Supplementary Table 1 The genes for F_1 pollen sterility and their located chromosomal substituted segments in the Taichung65 isogenic sterile lines (TISLs) used in this study

TISL	Donor	Target gene	Chromosome	Marker on substituted segments	The length of substituted segments (cM)
TISL-Xb	Xiobaidao	*Sb*	5	PSM341--PSM7-PSM8-(*Sb*)-PSM214-PSM202-PSM206--RM548	11.50
TISL-Db	Dijiaowujian	*Sb*	5	PSM341--PSM7-PSM8-(*Sb*)-PSM214-PSM202-PSM206--RM413	10.55
TISL-Ob	Oluenchung	*Sb*	5	PSM341--PSM7-PSM8-(*Sb*)-PSM214-PSM202-PSM206--RM413	10.55
TISL-Dc	Dijiaowujian	*Sc*	3	RM545--RM36-RM517-RM546-PSM428-PSM429-(*Sc*)-RM218--RM7	15.35
TISL-Pc	Pehku	*Sc*	3	RM545--RM36-RM517-RM546-PSM428-PSM429-(*Sc*)-RM218--RM7	15.35
TISL-Zc	Zaiyeqing	*Sc*	3	RM517--RM546-PSM428-PSM429-(*Sc*)-RM218--RM7	13.10
TISL-Gc	Guangluai4	*Sc*	3	RM545--RM36-RM517-RM546-PSM428-PSM429-(*Sc*)-RM218--RM7	15.35
TISL-Dd	Dijiaowujian	*Sd*	1	RM462--RM495-PSM23-PSM41-PSM43-PSM44-PSM12-PSM91-(*Sd*)-PSM80-RM84-RM323-RM1-RM283--PSM347	25.95
TISL-Zd	Zaiyeqing	*Sd*	1	PSM27--PSM26-PSM23-PSM41-PSM43-PSM44-PSM12-PSM91-(*Sd*)-PSM80-RM84-RM323-RM1-RM283-PSM347--RM522	20.60
TISL-Gd	Guangluai4	*Sd*	1	PSM23--PSM41-PSM43-PSM44-PSM12-PSM91-(*Sd*)-PSM80-RM84-RM323-RM220--RM1	12.85
TISL-Ge	Guangluai4	*Se*	12	RM415--PSM459-PSM181-PSM180-(*Se*)-PSM182-RM19--RM247	14.95

Supplementary Table 2 The F_1 pollen fertility and the F_2 distorted segregation from the crosses of TISLs with T65 (The second cropping season in 2011)

Gene for F_1 pollen sterility	TISL	F_1 pollen fertility (%±SD)	Marker tested	Ratio of genotypes in F_2 (*JJ*:*IJ*:*II*) [1)]	χ^2（1:2:1）
Sb	TISL-Xb	41.62±1.24	PSM8	3:81:70	58.71**[2)]
	TISL-Db	36.59±1.32		5:65:75	69.14**
	TISL-Ob	41.53±0.84		1:84:73	66.25**
	Mean	39.91±1.68		9:230:218	191.18**
Sc	TISL-Dc	49.14±0.50	RM218	3:74:78	72.90**
	TISL-Pc	54.45±0.67		4:77:79	70.54**
	TISL-Zc	55.46±0.59		3:88:68	54.96**
	TISL-Gc	51.19±0.53		2:83:75	66.84**
	Mean	52.56±1.46		12:322:300	261.81**
Sd	TISL-Dd	79.00±0.73	PSM12	25:83:52	9.34**
	TISL-Zd	69.92±0.41		11:83:63	34.96**
	TISL-Gd	74.65±0.79		23:87:50	10.34**
	Mean	74.52±2.63		59:253:165	48.87**
Se	TISL-Ge	45.62±0.96	PSM180	6:148:148	133.66**

1) *JJ*, genotypes of T65 (*S*-j/*S*-j); *IJ*, heterozygous genotypes (*S*-i/*S*-j); *II*, genotypes of TISLs (*S*-i/*S*-i).

2) **, significant difference at 0.01 level.

Supplementary Table 3 The pollen fertility in F_1 hybrids from the crosses of TISLs with *japonica* testers (%±SD, n=10, the second cropping season in 2011)

Locus	TISL	T65	Balilla	Akihikari	Zhonghua11	Nipponbare	Mean
Sb	TISL-Xb	41.62±1.24	11.01±0.71	35.93±1.13	14.51±0.70	25.49±0.84	25.71±5.91 A
	TISL-Db	36.59±1.32	22.62±0.72	35.75±0.35	19.71±0.34	33.58±1.02	29.65±3.53 B
	TISL-Ob	41.53±0.84	32.11±0.80	24.65±0.90	27.07±0.70	33.31±0.73	31.73±2.92 B
	Mean	39.91±1.66 C[1)]	21.91±6.10 A	32.11±3.73 B	20.43±3.64 A	30.79±2.65 B	29.03±3.58 (A)
Sc	TISL-Dc	49.14±0.50	45.29±0.82	36.50±0.53	46.34±0.95	50.21±0.66	45.50±2.42 A
	TISL-Pc	54.45±0.67	47.12±0.70	44.58±0.87	52.22±0.85	52.19±0.45	50.11±1.83 B
	TISL-Zc	55.46±0.59	47.57±0.64	49.47±1.01	46.93±0.81	50.86±0.48	50.06±1.52 B
	TISL-Gc	51.19±0.53	46.97±0.70	40.11±0.97	47.04±1.11	49.45±0.75	46.95±1.88 A
	Mean	52.56±1.46 D	46.74±0.50 B	42.67±2.81 A	48.13±1.37 BC	50.68±0.58 CD	48.15±1.70 (B)
Sd	TISL-Dd	79.00±0.73	76.55±0.93	65.10±0.94	77.96±0.87	84.08±0.78	76.54±3.13 C
	TISL-Zd	69.92±0.41	72.36±0.50	58.59±0.68	58.57±1.63	64.96±0.68	64.88±2.83 A
	TISL-Gd	74.65±0.79	75.90±0.97	66.21±0.73	56.98±0.87	79.36±1.11	70.62±4.04 B
	Mean	74.52±2.62 B	74.94±1.30 B	63.30±2.38 A	64.50±6.74 A	76.13±5.75 C	70.68±2.79 (C)

1) Multiple comparison method of analysis of variance is Duncan, different capital letters within the same row indication significant difference at 0.01 level. Letters in brackets reveal the significant difference of genetic effects among three loci on hybrid sterility.

Supplementary Table 4 The length of the substituted segments in the pyramiding line (TISL-Dbc-Gde)

Chr.	Target gene	Donor	Marker on the substituted segments	The length of substituted segments (cM)
1	*Sd*-i	Guangluai4	PSM23--PSM41-PSM43-PSM44-PSM12-PSM91-(*Sd*)-PSM80-RM84--RM1	11.25
3	*Sc*-i	Dijiaowujian	RM545--RM517-RM546-PSM428-PSM429-(*Sc*)-RM218--RM7	15.30
5	*Sb*-i	Dijiaowujian	PSM341--PSM8-(*Sb*)-PSM214-PSM202-PSM206-RM413--RM548	12.35
12	*Se*-i	Guangluai4	RM415--PSM459-PSM181-PSM180-(*Se*)-PSM182-RM19--RM247	14.95
11	no		RM254--PSM460--PSM346	5.10
11	no		RM286--PSM171--PSM172	3.10
Total				62.10

Supplementary Table 5 Comparisons of phenotypic values on some traits of TISL-Dbc-Gde and Taichung65 (T65) (mean ±SD, n=10)

Trait	First cropping season in 2012		Second cropping season in 2012	
	TISL-Dbc-Gde	T65	TISL-Dbc-Gde	T65
Day from sowing to heading	103.40±0.68[1)]	100.00±0.77	75.90±1.55	76.95±2.04
Plant height (cm)	121.32±5.29	124.43±3.84	112.65±4.66	113.57±4.98
1000-grain weight (g)	23.71±0.70	24.60±1.52	29.21±0.84	28.94±1.83
Grain length (mm)	7.15±0.10	7.02±0.17	7.14±0.09	7.04±0.13
Grain width (mm)	3.32±0.06	3.30±0.13	3.30±0.08	3.30±0.07
Panicle number per plant	8.33±1.21	8.17±0.75	8.17±0.98	8.33±1.21

1) Plant are grown in randomized complete block design, *t* test two sided reveals non-significant difference between TISL-Dbc-Gde and T65 on traits investigated at 0.05 level.

Supplementary Table 6 The pollen fertility and spikelet fertility in the F_1 hybrids between TISL-Dbc-Gde and testers (n=15, the second cropping season in 2011)

Tester	Pollen fertility (%±SD)	Spikelet fertility (%±SD)
japonica tester		
T65	1.58±0.73	Sterility
Balilla	1.17±0.63	Sterility
Akihikari	1.30±0.85	Sterility
Nipponbare	0.01±0.32	Sterility
Zhonghua11	0.81±0.94	Sterility
Mean	0.97±0.27	Sterility
indica tester		
Tetep	87.57±1.12	59.40±0.36
Amol3	87.38±1.02	71.63±0.53
9311	82.13±0.61	58.56±0.49
Aijiaonante	93.45±0.42	58.96±0.65
Guangluai4	90.58±0.86	57.69±0.77
Mean	88.22±1.89	61.25±2.61

Supplementary Table 7 The pollen fertility and spikelet fertility of the F_1 hybrids between TISL-Dbc-Gde or T65 and testers (%±SD, n=15, the second cropping season in 2012)

Tester	TISL-Dbc-Gde		T65	
	Pollen fertility	Spikelet fertility	Pollen fertility	Spikelet fertility
japonica tester				
Zhonghua11	2.07±0.37 **[1)]	1.38±0.88 **	91.34±0.45	94.30±1.09
Nipponbare	1.07±0.49 **	1.95±0.63 **	94.42±0.37	94.50±1.20
Akihikari	3.82±0.28 **	1.31±0.72 **	88.44±0.33	95.23±0.64
Balilla	2.96±0.15 **	1.53±0.86 **	90.48±0.57	95.68±1.42
T65	2.08±0.53 **	1.00±0.52 **	92.59±0.21	93.45±0.98
Mean	2.40±0.56 **	1.43±0.78 **	91.40±0.45	94.60±0.45
indica tester				
Aijiaonante	90.36±0.28 **	55.40±1.19 **	9.99±0.28	2.68±0.51
Guangluai4	88.81±0.34 **	57.44±0.48 **	20.91±0.48	6.09±0.35
IR36	88.46±0.40 **	55.84±0.52 **	22.62±0.34	12.11±0.14
9311	87.12±0.31	60.08±0.69	—	—
Mean	89.17±0.54 **	57.15±0.86 **	17.83±0.69	6.96±0.98

1) *t* test two sided is used to test the significance of difference between TISL-Dbc-Gde/tester and T65/tester on pollen fertility and spikelet fertility, ** indicating the significant difference at 0.01 level.

Supplementary Table 8 Segregation of the genes for F_1 sterility in F_2 populations from TISL-Dbc-Gde (P_1) and *indica* testers (P_2)

Gene for F_1 sterility	*indica* tester	Marker tested	No. of plants	Ratio of genotypes			χ^2（1:2:1）
				P_1P_1	P_1P_2	P_2P_2	
Sa	Tetep	G02-148	160	42	84	34	1.20
	Amol3	G02-148	159	54	74	31	7.42*[1)]
	9311	G02-148	159	41	81	37	0.26
	Aijiaonante	G02-148	160	53	70	37	5.70
	Guangluai 4	G02-148	160	55	71	34	7.54*
	Mean		798	245	380	173	14.80**
Sb	Tetep	PSM8	160	42	86	32	2.15
	Amol3	RM413	159	36	82	41	0.47
	9311	PSM8	160	32	85	43	2.14
	Guangluai 4	PSM8	160	35	86	39	1.10
	Mean		639	145	339	155	2.69
Sc	Amol3	RM7	160	33	88	39	2.05
	9311	RM218	158	42	78	38	0.03
	Aijiaonante	RM7	157	41	79	37	0.21
	Mean		475	116	245	114	0.49
Sd	Tetep	PSM12	160	42	84	34	1.20
	Amol3	PSM12	157	42	73	42	0.77
	9311	PSM12	159	64	67	28	20.23**
	Aijiaonante	PSM12	160	34	76	50	3.60
	Guangluai 4	PSM12	160	38	76	46	1.20
	Mean		796	220	376	200	3.44
Se	Tetep	PSM180	160	35	82	43	0.90
	Amol3	IND19	158	41	76	41	0.23
	9311	IND19	159	40	78	41	0.07
	Aijiaonante	IND19	160	39	82	39	0.10
	Mean		637	155	318	164	0.26
S5	Tetep	RM50	159	18	70	71	37.60**
	Amol3	RM276	160	30	76	54	7.60*
	9311	RM276	160	18	70	72	38.95**
	Aijiaonante	RM276	158	6	90	62	42.76**
	Guangluai 4	RM276	160	10	86	64	37.35**
	Mean		797	82	392	323	145.96**

1) *&**, significant difference at 0.05and 0.01 levels, respectively.

Supplementary Table 9 Genotypes at the *S5* locus in 171 accessions of *O. sativa*

Type	*S5* locus				No. of accesions
	ORF3	*ORF4*	*ORF5*	Genotype	
1	+	+	+	*S5*-i/*S5*-i	33
2	+	-	+	*S5*-i/*S5*-i	57
3	-	+	-	*S5*-j/*S5*-j	64
4	+	+	n	*S5*-n/*S5*-n	13
5	+	-	n	*S5*-n/*S5*-n	2
6	-	-	n	*S5*-n/*S5*-n	2
Toatl					171

Supplementary Table 10 Substituted segments with genes for F_1 sterility in *indica*-compatible *japonica* lines (ICJLs)

ICJL	Chr.	Donor	Gene for F_1 sterility	Marker on substituted segments	The length of substituted segments (cM)
ICJL-T-W6	1	Guangluai4	*Sd*-i	PSM23--PSM41-PSM43-PSM44-PSM12-PSM91-(*Sd*)-PSM80-RM84--RM1	11.25
	12	Guangluai4	*Se*-i	RM415--PSM459-PSM181-PSM180-(*Se*)-PSM182--RM247	14.95
	3	Dijiaowuian	*Sc*-i	RM545--RM546-PSM428-PSM429-(*Sc*)-RM218--RM7	15.30
	5	Dijiaowuian	*Sb*-i	PSM341--PSM8-(*Sb*)-PSM214-PSM202--RM413	8.55
	6	Katy	*S5*-n	RM204--PSM349-IND5-(*S5*)-RM276--RM50	11.10
ICJL-T-W19	1	Guangluai4	*Sd*-i	PSM23--PSM41-PSM43-PSM44-PSM12-PSM91-(*Sd*)-PSM80-RM84--RM1	11.25
	12	Guangluai4	*Se*-i	RM415--PSM459-PSM181-PSM180-(*Se*)-PSM182--RM247	14.95
	3	Dijiaowuian	*Sc*-i	RM545--RM546-PSM428-PSM429-(*Sc*)-RM218--RM7	15.30
	5	Dijiaowuian	*Sb*-i	PSM341--PSM8-(*Sb*)-PSM214-PSM202-PSM206--RM413	9.50
	6	Kyeema	*S5*-n	RM204--RM225-PSM349-IND5-(*S5*)-RM276--RM50	20.85
ICJL-T-W21	1	Guangluai4	*Sd*-i	PSM23--PSM41-PSM43-PSM44-PSM12-PSM91-(*Sd*)-PSM80-RM84--RM1	11.25
	12	Guangluai4	*Se*-i	RM415--PSM459-PSM181-PSM180-(*Se*)-PSM182--RM247	14.95
	3	Dijiaowuian	*Sc*-i	RM545--RM517-RM546-PSM428-PSM429-(*Sc*)-RM218--RM7	15.30
	5	Dijiaowuian	*Sb*-i	PSM341--PSM8-(*Sb*)-PSM214-PSM202-PSM205--RM413	9.50
	6	IR65598-112-2	*S5*-n	RM225--PSM349-IND5-(*S5*)-RM276--RM50	10.95
ICJL-T-W22	1	Guangluai4	*Sd*-i	PSM23--PSM41-PSM43-PSM44-PSM12-PSM91-(*Sd*)-PSM80-RM84--RM1	11.25
	12	Guangluai4	*Se*-i	RM415--PSM459-PSM181-PSM180-(*Se*)-PSM182--RM247	14.95
	3	Dijiaowuian	*Sc*-i	RM545--RM517-RM546-PSM428-PSM429-(*Sc*)-RM218--RM7	15.30
	5	Dijiaowuian	*Sb*-i	PSM341--PSM8-(*Sb*)-PSM214-PSM202-PSM206--RM413	9.50
	6	Khazar	*S5*-n	RM204--PSM349-IND5-(*S5*)-RM276--RM50	11.10
ICJL-T-W23	1	Guangluai4	*Sd*-i	PSM23--PSM41-PSM43-PSM44-PSM12-PSM91-(*Sd*)-PSM80-RM84--RM1	11.25
	12	Guangluai4	*Se*-i	RM415--PSM459-PSM181-PSM180-(*Se*)-PSM182--RM247	14.95
	3	Dijiaowuian	*Sc*-i	RM545--RM517-RM546-PSM428-PSM429-(*Sc*)-RM218--RM7	15.30
	5	Dijiaowuian	*Sb*-i	PSM341--PSM8-(*Sb*)-PSM214-PSM202-PSM205--RM413	9.50
	6	Lemont	*S5*-n	RM204--RM225-PSM349-IND5-(*S5*)-RM276-RM50--RM557	24.20
ICJL-T-W24	1	Guangluai4	*Sd*-i	PSM23--PSM41-PSM43-PSM44-PSM12-PSM91-(*Sd*)-PSM80-RM84--RM1	11.25
	12	Guangluai4	*Se*-i	RM415--PSM459-PSM181-PSM180-(*Se*)-PSM182--RM247	14.95
	3	Dijiaowuian	*Sc*-i	RM545--RM517-RM546-PSM428-PSM429-(*Sc*)-RM218--RM7	15.30
	5	Dijiaowuian	*Sb*-i	PSM341--PSM8-(*Sb*)-PSM214-PSM202--RM413	8.55
	6	Starbonnet99	*S5*-n	RM225--PSM349-IND5-(*S5*)-RM276--RM50	10.95
ICJL-T-W27	1	Guangluai4	*Sd*-i	PSM23--PSM41-PSM43-PSM44-PSM12-PSM91-(*Sd*)-PSM80-RM84--RM1	11.25
	12	Guangluai4	*Se*-i	RM415--PSM459-PSM181-PSM180-(*Se*)-PSM182--RM247	14.95
	3	Dijiaowuian	*Sc*-i	RM545--RM517-RM546-PSM428-PSM429-(*Sc*)-RM218--RM7	15.30
	5	Dijiaowuian	*Sb*-i	PSM341--PSM8-(*Sb*)-PSM214-PSM202--RM413	8.55
	6	IAPAR9	*S5*-n	RM204--RM225-PSM349-IND5-(*S5*)-RM276--RM50	20.85

Supplementary Table 11 The fertility of the F_1 hybrids from the crosses between ICJLs and testers (%±SD, n=10, the first cropping season in 2014)

Line	Pollen fertility					Spikelet fertility				
	japonica tester		*indica* tester			*japonica* tester		*indica* tester		
	T65	Sanlicun	Guangluai4	Amol3	Tetep	T65	Sanlicun	Guangluai4	Amol3	Tetep
ICJL-T-W6	3.56±0.68	2.13±0.23	90.34±0.55	90.45±0.26	91.32±0.79	1.23±0.42	2.03±0.40	89.34±0.58 **[1)]	99.32±0.43**	88.00±0.33**
ICJL-T-W19	3.32±0.74	2.15±1.24	87.56±0.61	87.89±0.70	89.43±0.38	1.45±0.25	3.24±0.49	89.34±1.26**	89.87±0.85**	85.01±0.71**
ICJL-T-W21	2.41±0.75	3.17±0.77	90.67±0.33	88.34±0.67	87.43±0.22	1.54±0.23	2.43±0.29	93.21±0.79**	90.32±0.74**	87.00±0.25**
ICJL-T-W23	3.56±0.81	3.15±0.39	89.56±0.93	91.34±0.58	90.87±0.35	1.23±0.92	2.00±1.30	90.67±0.52**	92.56±0.76**	89.01±0.62**
ICJL-T-W24	4.56±0.49	1.35±0.66	87.23±0.37	90.56±0.91	91.23±0.26	1.76±0.87	1.45±0.35	92.41±0.49**	91.65±0.75**	84.00±0.77**
TISL-Dbc-Gde (CK)	3.24±0.80	2.95±0.39	88.45±0.45	89.65±0.60	89.67±0.54	2.01±0.48	1.57±0.81	57.34±0.83	69.32±0.72	44.78±0.35

1) *t* test two sided is used to test the significance of difference between ICJL/tester and TISL-Dbc-Gde/tester on pollen fertility and spikelet fertility, ** indicating the significant difference at 0.01 level.

Supplementary Table 12 The pollen fertility of the F_1 hybrids from the crosses between ICJLs and testers (%±SD, n=10, the second cropping season in 2014)

Line	*indica* tester					*japonica* tester					
	Guangluai4	Aijiaonante	Xiangyazhan	9311	Mean	Balilla	Yerua	Sanlicun	Shennong265	T65	Mean
ICJL-T-W6	-[1)]	-	86.84±0.45	87.95±0.34	87.40±0.39 B[2)]	-	-	1.47±0.30	-	2.36±1.40	1.92±0.44 A
ICJL-T-W19	88.84±0.79	-	-	86.93±0.34	87.89±0.68 B	-	2.57±0.41	-	-	1.92±0.61	2.25±0.27 A
ICJL-T-W21	-	-	-	88.09±0.65	88.09±0.65 B	-	-	1.22±0.41	-	2.19±0.43	1.71±0.40 A
ICJL-T-W22	90.33±0.89	93.91±0.34	-	-	92.12±1.27 B	0.95±0.77	-	1.16±0.84	3.80±0.39	2.27±0.61	2.05±0.65 A
ICJL-T-W23	-	92.96±0.33	87.34±0.33	87.80±0.67	89.37±1.47 B	2.08±0.66	-	1.33±0.48	3.66±0.64	3.55±0.51	2.66±0.57 A
ICJL-T-W24	-	-	89.07±0.37	90.00±0.51	89.54±0.33 B	-	-	1.21±0.27	-	3.49±0.33	2.35±1.14 A
ICJL-T-W27	89.15±0.26	93.79±0.33	-	89.00±0.20	90.65±1.28 B	1.94±0.20	-	0.97±0.32	2.52±0.46	3.42±0.29	2.21±0.59 A
TISL-Dbc-Gde (CK1)	-	92.93±0.68	88.14±0.38	85.69±0.73	88.92±1.36 B	2.44±0.51	1.40±0.39	0.96±0.46	4.00±0.81	-	2.20±0.54 A
T65 (CK2)	23.64±0.25	7.71±0.36	-	4.57±0.34	11.97±1.50 A	92.96±0.07	93.19±0.59	72.26±0.46	80.21±0.42	94.54±0.26	86.63±0.60 B

1) -, No data.

2) The least significant difference (LSD) method is used to compare the means between ICJLs/testers and CK1 or CK2/testers on pollen fertility. Different capital letters within the same column indicate significant difference at 0.01 level.

Supplementary Table 13 The spikelet fertility of the F_1 hybrids from the crosses between ICJLs and testers (%±SD, n=10, the second cropping season in 2014)

Line	*indica* tester					*japonica* tester					
	Guangluai4	Aijiaonante	Xiangyazhan	9311	Mean	Balilla	Yerua	Sanlicun	Shennong265	T65	Mean
ICJL-T-W6	-[1)]	-	87.99±0.52	89.65±0.52	88.82±0.83 C[2)]	-	-	3.19±0.57	-	2.48±0.71	2.84±0.36 A
ICJL-T-W19	92.41±0.61	-	-	90.88±0.38	91.65±0.77 C	-	3.94±0.51	-	-	1.91±0.45	2.93±0.83 A
ICJL-T-W21	-	-	-	88.70±0.52	88.70±0.52 C	-	-	2.31±0.70	-	2.24±0.30	2.28±0.03 A
ICJL-T-W22	89.97±0.65	91.34±0.26	-	-	90.66±0.68 C	2.34±0.83	-	3.41±1.53	2.17±0.58	1.88±0.68	2.45±0.33 A
ICJL-T-W23	-	89.56±0.65	88.83±0.69	87.94±0.24	88.78±0.47 C	1.91±1.75	-	3.37±0.42	2.37±0.52	1.77±0.63	2.36±0.36 A
ICJL-T-W24	-	-	89.52±0.33	89.51±0.62	89.52±0.01 C	-	-	2.89±1.06	-	2.15±0.47	2.52±0.37 A
ICJL-T-W27	91.72±0.68	91.18±0.33	-	88.98±0.39	90.63±0.84 C	2.47±0.54	-	2.64±0.62	1.70±0.53	1.93±0.43	2.19±0.26 A
TISL-Dbc-Gde (CK1)	-	61.19±0.78	56.18±0.42	59.02±0.35	58.80±0.47 B	1.76±0.85	4.72±0.60	2.51±0.76	2.50±0.50	-	2.87±0.64 A
T65 (CK2)	7.41±0.53	5.73±0.96	-	9.33±0.50	7.49±0.90 A	93.11±0.69	92.80±0.96	92.30±0.54	93.57±0.77	94.06±0.53	93.17±0.30 B

1) -, No data.

2) The least significant difference (LSD) method is used to compare the means between ICJLs/testers and CK1 or CK2/testers on spikelet fertility. Different capital letters within the same column indicate significant difference at 0.01 level.

Supplementary Table 14 Primer sequences of the markers developed in our laboratory

Marker name	Chromosome	Forward primer	Reverse primer	Marker type[1)]
PSM8	5	CCAggTCACCACCACAAT	AAATCCgAATCgCATCAg	SSR
PSM12	1	gTCAggAgACTTggTTTTgAA	AggTgATgCTggAAgAATAgA	SSR
PSM180	12	AAAACCTTgCTCAgCAggAC	gCACCACATTTggTTTCCTT	SSR
IND19	12	ACATTgCggTATTATCTg	AACAgAggCACATTTCAT	InDel
ID5	6	gAAAgAAAgAAgggATTAAATTTgC	TTCgTgAATgTCATACTgTgCTT	InDel

1) InDel: insertion/deletion.

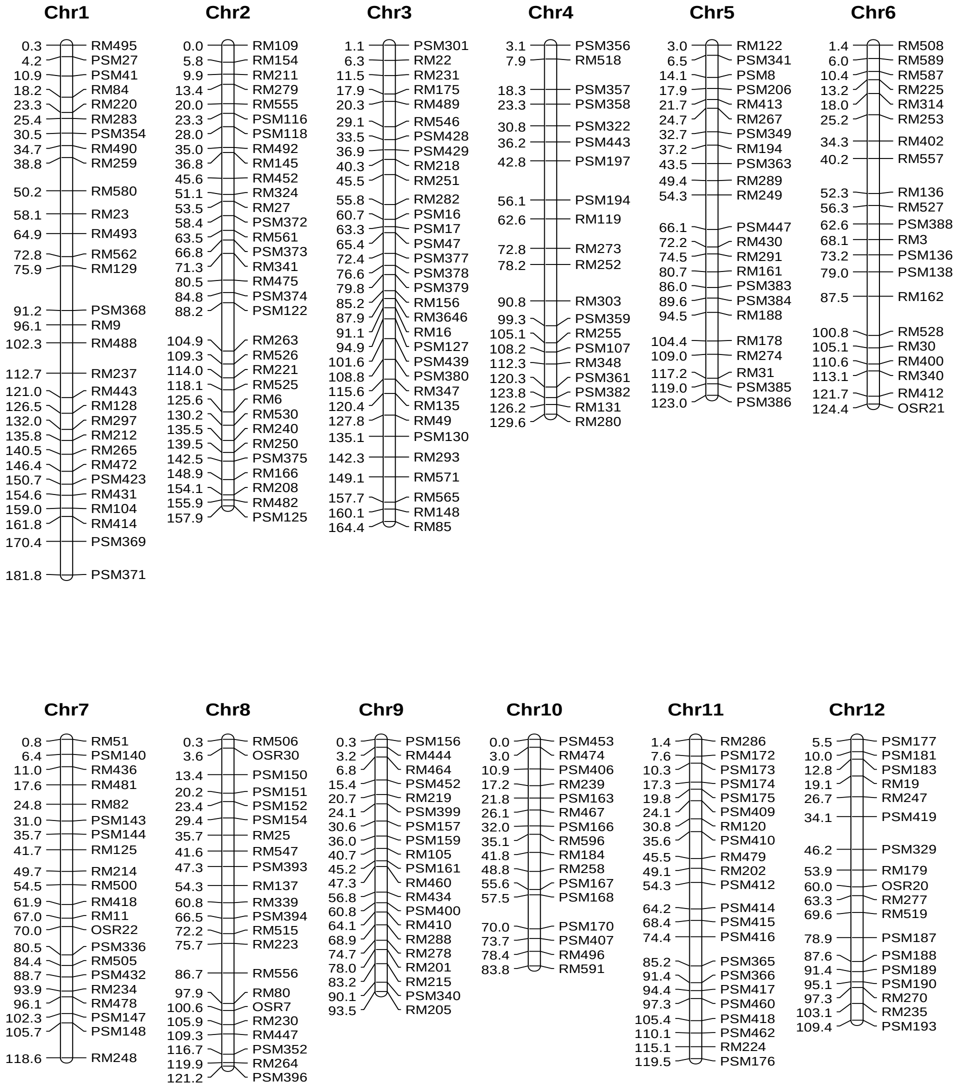

Supplementary Figure 1 Distribution of polymorphic markers between T65 and six donors on genetic map of rice
